# Bioinformatic Analyses of the Ferroptosis-Related lncRNAs Signature for Ovarian Cancer

**DOI:** 10.3389/fmolb.2021.735871

**Published:** 2022-01-18

**Authors:** Jianfeng Zheng, Jialu Guo, Yahui Wang, Yingling Zheng, Ke Zhang, Jinyi Tong

**Affiliations:** ^1^ Department of Obstetrics and Gynecology, Affiliated Hangzhou Hospital, Nanjing Medical University, Hangzhou, China; ^2^ Department of Obstetrics and Gynecology, Hangzhou Women’s Hospital, Hangzhou, China; ^3^ Department of Radiation Oncology, Hangzhou Cancer Hospital, Hangzhou, China

**Keywords:** ovarian cancer, ferroptosis, lncRNA, risk model, immune

## Abstract

Both ferroptosis and lncRNAs are significant for ovarian cancer (OC). Whereas, the study of ferroptosis-related lncRNAs (FRLs) still few in ovarian cancer. We first constructed an FRL-signature for patients with OC in the study. A total of 548 FRLs were identified for univariate Cox regression analysis, and 21 FRLs with significant prognosis were identified. The prognostic characteristics of nine FRLs was constructed and validated, showing opposite prognosis in two subgroups based on risk scores. The multivariate Cox regression analysis and nomogram further verified the prognostic value of the risk model. By calculating ferroptosis score through ssGSEA, we found that patients with higher risk scores exhibited higher ferroptosis scores, and high ferroptosis score was a risk factor. There were 40 microenvironment cells with significant differences in the two groups, and the difference of Stromal score between the two groups was statistically significant. Six immune checkpoint genes were expressed at different levels in the two groups. In addition, five m6A regulators (FMR1, HNRNPC, METTL16, METTL3, and METTL5) were higher expressed in the low-risk group. GSEA revealed that the risk model was associated with tumor-related pathways and immune-associated pathway. We compared the sensitivity of chemotherapy drugs between the two risk groups. We also explored the co-expression, ceRNA relation, cis and trans interaction of ferroptosis-related genes and lncRNAs, providing a new idea for the regulatory mechanisms of FRLs. Moreover, the nine FRLs were selected for detecting their expression levels in OC cells and tissues.

## Introduction

Approximately 150,000 women die of ovarian cancer (OC) every year, making it the highest death rate among gynecological tumors (Lheureux et al., 2019). The development of OC is regulated by many cytokines and signaling pathways (Narod, 2016). Although the prognosis for early-stage cancer patients is better, the vast majority of patients are already in the advanced stage when they are first diagnosed (Siegel et al., 2020). Hence, the current urgent problem to be solved is to improve the prognosis of OC patients.

Ferroptosis is a process that regulates cell death and its particularity is lipid peroxide in cell membrane in an iron-dependent manner, which is different from apoptosis and necrosis (Yang and Stockwell, 2008). The key to ferroptosis lies in the production of reactive oxygen species in Fenton reaction and the reduction of glutathione-dependent peroxidase 4 (GPX4) (Xie et al., 2016; Yang and Stockwell, 2016). Ferroptosis is correlated with a variety of biochemical processes and can inhibit the proliferation of tumor tissues by depriving iron in cancer cells or changing the metabolism of iron ions in tumor tissues (Mou et al., 2019; Xu et al., 2019; Wang et al., 2020a; Wu et al., 2020). Studies have shown that continuous iron stimulation is one of the high-risk factors for the occurrence and development of OC(Lattuada et al., 2015) and ferroptosis is considered as a potential therapeutic target for OC (Lin and Chi, 2020).

The length of lncRNAs is more than 200 nucleotides and lncRNAs are involved in carcinogenesis or suppression in a variety of cancers, including OC(Wang et al., 2019a; Braga et al., 2020). Studies have shown that lncRNAs can regulate ferroptosis in tumor cells at the transcriptional or post-transcriptional level, and its mechanisms involve glutamine decomposition, mitochondria associated proteins, iron metabolism, glutathione metabolism, lipid peroxidation, and p53 signaling pathways (Jiang et al., 2021). However, there are few studies on the mechanism of ferroptosis-related lncRNA (FRL) in OC pathology.

In our study, nine optimized FRLs were identified. The prognostic signature of nine FRLs was constructed and validated, showing opposite prognosis in two subgroups based on risk scores. In addition, we identified differences in immune microenvironment, m6A regulatory factors, immune checkpoints, and sensity to several chemotherapeutic agents between risk groups. We also explored the co-expression, ceRNA relation, cis and trans interaction of ferroptosis-related genes and lncRNAs, providing a new idea for the regulatory mechanisms of FRLs. Furthermore, the nine FRLs were selected for detecting their expression levels.

## Materials and Methods

### Data Collection

The processed RNA sequencing profiles (UCSC TOIL RNA-seq) with clinical information were extracted from TCGA(Goldman et al., 2020). There were 416 OC samples and 88 normal samples in our study. After converting Ensemble Gene to Gene Symbol and filtering through genes with low expression, the lncRNAs were identified according to the annotation information in GENCODE database (Harrow et al., 2012). We obtained 288 ferroptosis-related genes (FRGs), of which 108 FRGs were drivers, 69 FRGs were suppressors and 111 FRGs were markers, from FerrDb database (Zhou et al., 2020). In the aggregate, 215 mRNAs associated with ferroptosis were screened via matching it to the filtered mRNAs above. Differential analysis of ferroptosis-related mRNAs and annotated lncRNAs between tumor and normal samples was according to linear regression and Empirical Bayes (*p* < 0.05, |logFC|>1) (Ritchie et al., 2015). In addition, we used the Benjamini and Hochberg for multiple testing correction to get adjusted *p* value and assessed the differences at multiple and significance levels (p-adjusted<0.05, |logFC|>1) (Ferreira, 2007; Ghosh, 2019). We calculated the Pearson correlation coefficient between FRGs and lncRNAs to filter the lncRNAs that are significantly related to mRNAs (|r|>0.5, *p* < 0.001).

### Bioinformatic Analysis

According to R’s survival package (Wang et al., 2016), univariate Cox regression was performed on candidate FRLs to screened prognostic lncRNAs. The R-language random function was used to randomly divide the total OC samples (total set, TOS) into two sets, training set (TS) and validation set (VS). Whereafter, using the LASSO Cox regression (Tibshirani, 1997)in the glmnet software package of R (Engebretsen and Bohlin, 2019)and 20 times cross-validation analyses, the optimal combination of FRL markers was screened based on the TS set. We constructed a risk model for OC patients based on formula:

$$\mathrm{Risk}\mathrm{score}\left(\mathrm{RS}\right)=\sum \beta_{1ncRNA}\times {\mathrm{EXP}}_{1ncRNA}$$

In the formula, *ß*_lncRNA_ represented the Lasso prognostic coefficient for optimized FRLs and Exp_lncRNA_ meant the expression level. In the TS set, the OC samples were classified into high-risk (with a RS higher than or equal to the median value of RSs, *n* = 104) and low-risk (with a RS lower than the median value of RSs, *n* = 104) according to the mean value of RSs. In addition, in order to verify the accuracy of the model, RS values of each sample in TOS (high-risk: *n* = 208; low-risk: *n* = 208) and VS (high-risk: *n* = 104; low-risk: n = 104) sets were calculated using the same regression coefficient. The association between the risk groups and the prognosis statuses was evaluated by using the Kaplan-Meier curve method of survival package in R.

A visual nomogram was constructed after univariate and multivariate Cox regression analysis of clinical features and risk groups, and verified by correction curves to determine the accuracy of the risk model.

Based on ssGSEA algorithm, we calculated the enrichment fraction of FRGs in different samples to obtain the ferroptosis score (FS). Based on the median of FSs, the samples were divided into high-score (*n* = 208) and low-score (*n* = 208) groups. We compared the differences in survival between patients with high-score and low-score and, in conjunction with the risk scores, we also analyzed survival among patients in different subgroups (low-risk + low-score: *n* = 104; low-risk + high-score: *n* = 104; high-risk + low-score: *n* = 104; high-risk + high-score: *n* = 104). DAVID was used to analyze the biological functions of potential FRGs (Huang et al., 2009a; Huang et al., 2009b). Differentially expressed genes (DEGs, |logFC| and FDR<0.05) were filtered between the two risk groups on the basis of the limma R package (Clark et al., 2014). Candidate small molecular drugs and mechanisms of action were predicted via uploading DEGs to CMAP database (Lamb et al., 2006; Lamb, 2007).

We used five algorithms (Estimate (Yoshihara et al., 2013), ssGSEA (Jia et al., 2018); (Hänzelmann et al., 2013), CIBERSORT (Newman et al., 2015; Charoentong et al., 2017), MCPcounter (Shi et al., 2020), and xCell (Aran et al., 2017)) to estimate differences in immune microenvironment between risk groups. The expression levels of immune checkpoint genes were extracted, and the expression differences among groups were compared by inter-group T test.

FRG-FRL co-expression (|PCC|>5, *p* < 0.001) network was constructed and visualized by Cytoscape3.6.1 (Shannon et al., 2003). The miRWalk speculated that the ferroptosis-related mRNAs targeted by the corresponding miRNAs (Dweep and Gretz, 2015). Furthermore, we combined the results of six commonly used databases (Microt4, miRWalk, miRDB, miRanda, Targetscan and RNA22) to obtain the miRNA-FRG relationship pairs. The miRNAs targeted by corresponding FRLs of the risk model were speculated by miranda (Enright et al., 2003). FRLs and FRGs regulated by the same miRNA were defined as ceRNAs mutually. The UCSC data set (Yuan et al., 2019) searched for potential *cis*-interactions between FRLs and mRNAs transcribed from the same chromosome within 200 kb. Then, further construct a network based on overlapped TFs to detect the *trans*-regulatory function of FRLs and FRGs (Shu et al., 2019).

Moreover, we assessed the Tumor Immune Dysfunction and Exclusion (TIDE) score through the TIDE database to predict the response to Immune Checkpoint Blockade (Fu et al., 2020). The chemotherapy drugs were extracted from Genomics of Drug Sensitivity in Cancer (GDSC) database (Yang et al., 2013) and the pRRophetic R package is used to calculate the 50% maximum inhibitory concentration (IC50) (Geeleher et al., 2014).

### Ethics Statement

With the approval of the Ethics Committee, specimens were taken from 30 patients. Fresh specimens (≤0.5 cm) were rapidly immersed in RNA Stabilization Solution (GenePharma, China) and incubated the samples overnight at 4°C. The tissue was completely permeated and then transferred to a -80 °C refrigerator for subsequent experiments.

### Real Time qPCR Analysis

Using ultrasonic crusher, the tissues were cracked by lysate. Total RNA was extracted from OC cells and tissues by using RNA Extraction Kit (TAKARA, Japan). Moreover, cDNA was gained after reverse transcription by using the PrimeScriptTMRT (TAKARA, Japan). PCR was performed by ABI 7500 instrument to evaluate the expression level of the nine FRLs by using TB Green® Premix Ex TaqTM (TAKARA, Japan). Total 1 μL cDNA was used as the template in a final 10 μL PCR reaction volume containing 5 μL TB Green Premix EX Taq, 0.4 μL corresponding primers (GenePharma, China), 0.2ul ROX Reference Dye II and 3.4 μL DEPC water. PCR was run as follows: 95.0 °C for 5s, 40 cycles of 95.0 °C for 5s and 60.0 °C for 34s. The melting curve was obtained at 95.0 °C for 15s, 60.0 °C for 1min and 95.0 °C for 15s. The primer sequences are shown in Supplementary Table S1.

### Statistical Analysis

We used R software package, TBtools, and GraphPad Prism for statistical analysis and visualization. Log-rank test was used to evaluate the statistical significance of the difference between the survival curves of two subgroups (Bland and Altman, 2004). Wilcoxon test was used to compare the differences between the two groups. The experiment was made in triplicate, and each experiment was repeated three times. Student’s T test or ANOVA were used for the experimental data. *p* < 0.05 was considered statistically significant.

## Results

**FIGURE 1:**
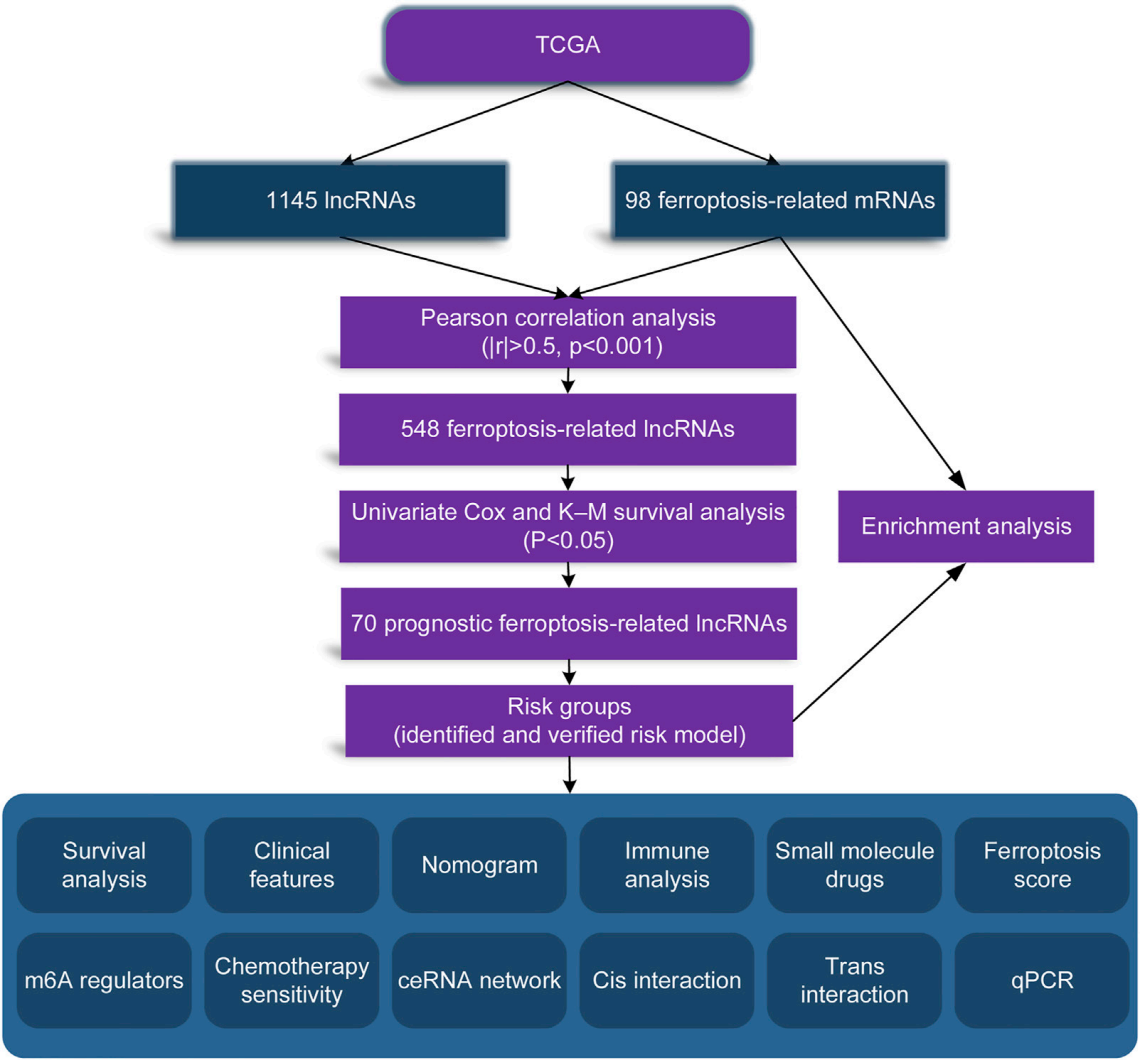
Flow diagram of our study.

In order to make our entire study facilitate to understanding, we made a flow chart, as shown in Figure 1.

### Differential Analysis

**FIGURE 2:**
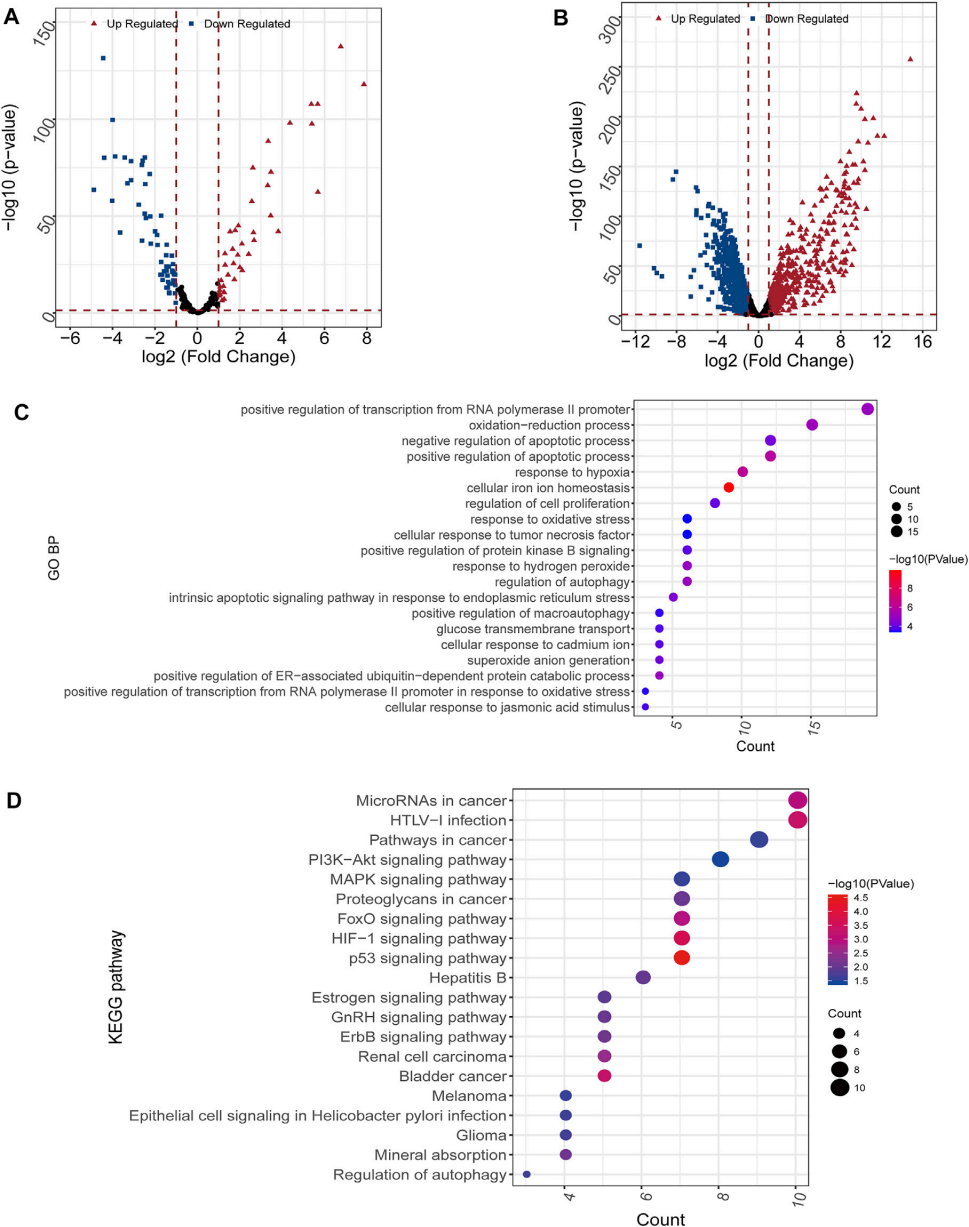
Differential and enrichment analysis **(A,B)** Volcano map of differentially expressed mRNAs **(A)** and lncRNAs **(B)**. Red triangle: up regulated; blue square: down regulated **(C)** GO analysis. The color scale represented *p* value and the circle size indicated count **(D)** KEGG analysis. The color scale represented *p* value and the circle size indicated count.

A total of 98 differential FRGs (Figure 2A) and 1,145 exegetical lncRNAs were identified (Figure 2B). Enrichment analysis further exhibited that 131 GO and 24 KEGG pathways were enriched based on aforementioned 98 FRGs. The result showed that the identified FRGs were closely related to several important biological processes or pathways, such as cellular response to tumor necrosis factor (Figure 2C), response to hypoxia (Figure 2C), PI3K-Akt/MAPK/HIF-1/p53 signaling pathway (Figure 2D), etc.

### Construction and Validation of the Predictive Signature

**FIGURE 3:**
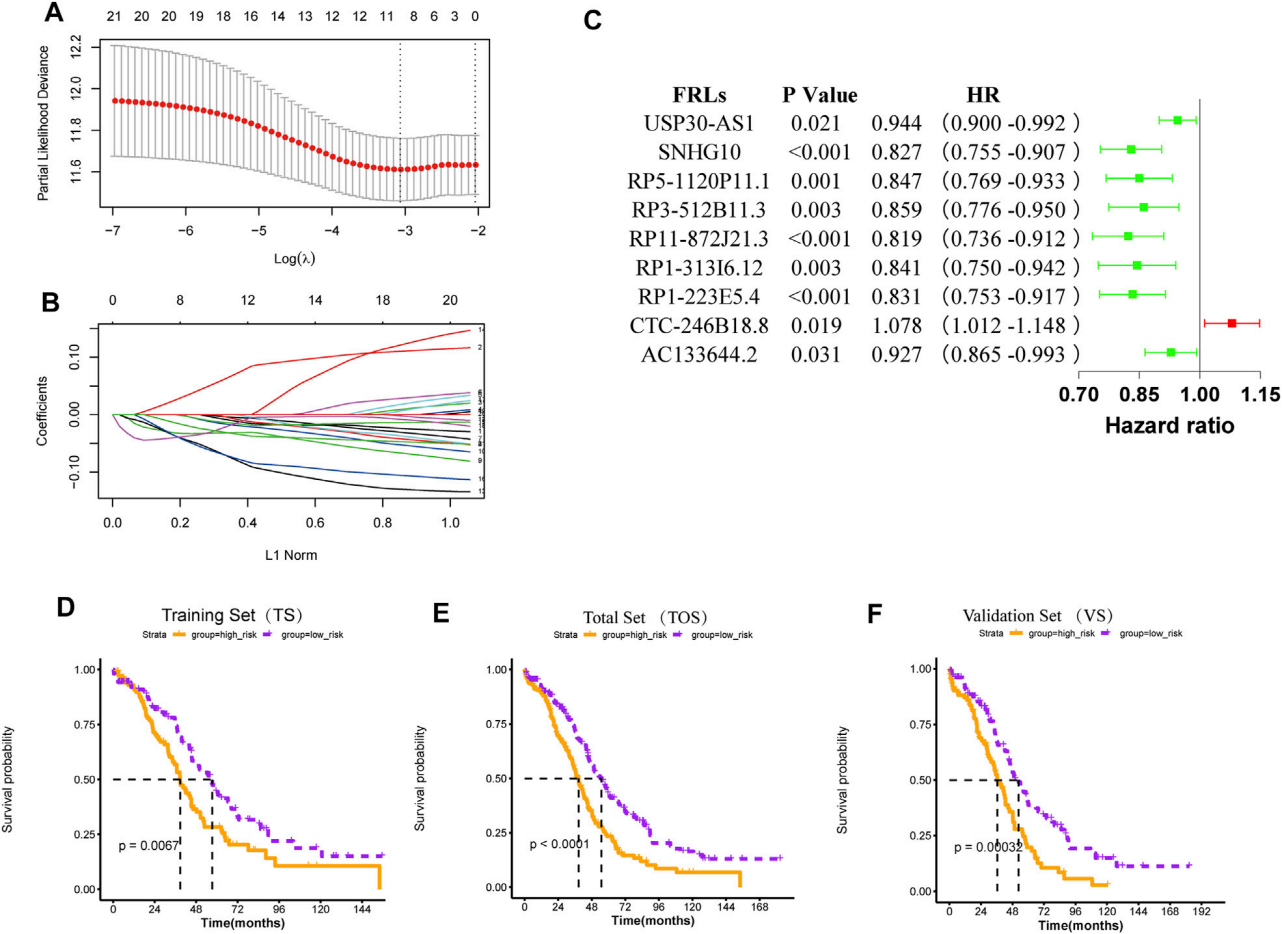
Construction and validation of the risk model **(A)** *λ* selection diagram. The two dotted lines indicated two particular values of *λ*. The left side was λ_min_ and the right side was λ_1se_. The λ_min_ was selected to build the model for accuracy in our study **(B)** LASSO Cox analysis **(C)** Forest plot of the prognostic ability of the nine optimal FRLs **(D-F)** The K-M survival curves of training set **(D)**, total set **(E)**, and validation set **(F)**.

548 FRLs were obtained by Pearson correlation analysis (|PCC|>0.5, *p* < 0.001) and FRL with significant prognosis was mined (*p* < 0.05). The FRL-signature was constructed by the LASSO Cox analysis of 70 prognostic FRLs. The *λ* selection diagram is shown in Figures 3A,B.

For 70 prognostic FRLs obtained, the LASSO Cox Regression model from glmnet package and 20-fold cross-validation analysis were further used to screen the optimal biomarker combinations of FRLs. Nine optimal FRLs were filtrated and FRL CTC-246B18.8 was a risk factor with HR > 1, while FRLs AC133644.2, RP1-223E5.4, RP1-313I6.12, RP11-872J21.3, RP3-512B11.3, RP5-1120P11.1, SNHG10, and USP30-AS1 were protective factors with HR < 1 (Figure 3C, Supplementary Figure S1). To further validate the expression levels of the nine FRLs in OC, the qPCR analysis was detected in our collected specimens and cells. The results showed that FRL CTC-246B18.8 was significantly upregulated in OC cells and samples (Supplementary Figures S2,3), while FRLs SNHG10 and USP30-AS1 were downregulated compared with normal control (Supplementary Figures S2,3).

**FIGURE 4:**
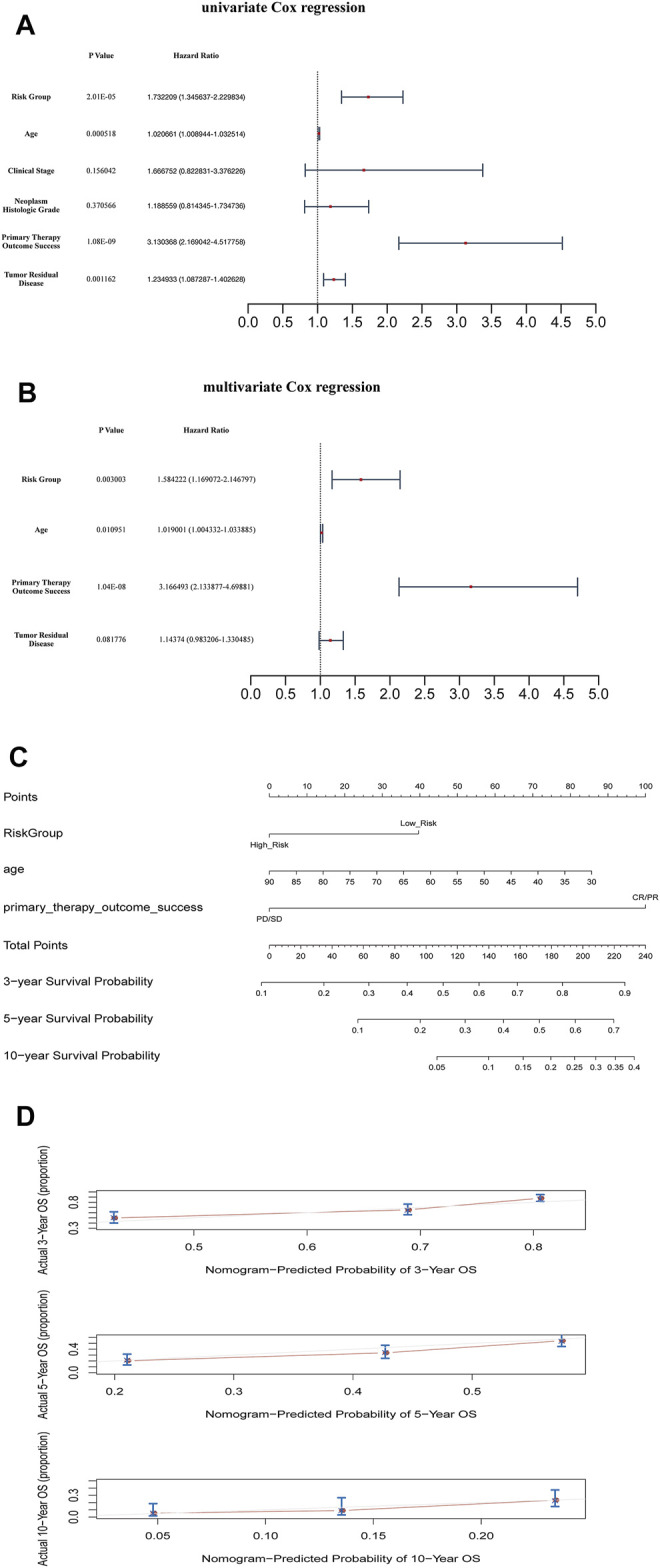
Clinical value of risk score by independent prognostic analysis **(A)** The univariate Cox regression analysis **(B)** The Cox regression analysis **(C)** The Nomogram model based on risk model and clinical features **(D)** The calibration plots of the nomogram. The closer it was to 45 degrees or the gray lines in the graph, the better the fitting effect.

According to the mean value of RSs, the patients with OC from TCGA database were divided into two risk subgroups. The K-M survival curves indicated a worse prognosis in the high-risk group (Figures 3D–F). The univariate and multivariate Cox regression analysis of clinical features and the risk model showed that age, “primary therapy outcome success”, and risk model were independent prognostic factors (Figures 4A,B). A nomogram was further constructed based on age, “primary therapy outcome success”, and risk model (Figure 4C). The calibration curve was drawn to prove the accuracy of the model (Figure 4D).

### Ferroptosis Score Analysis

**FIGURE 5:**
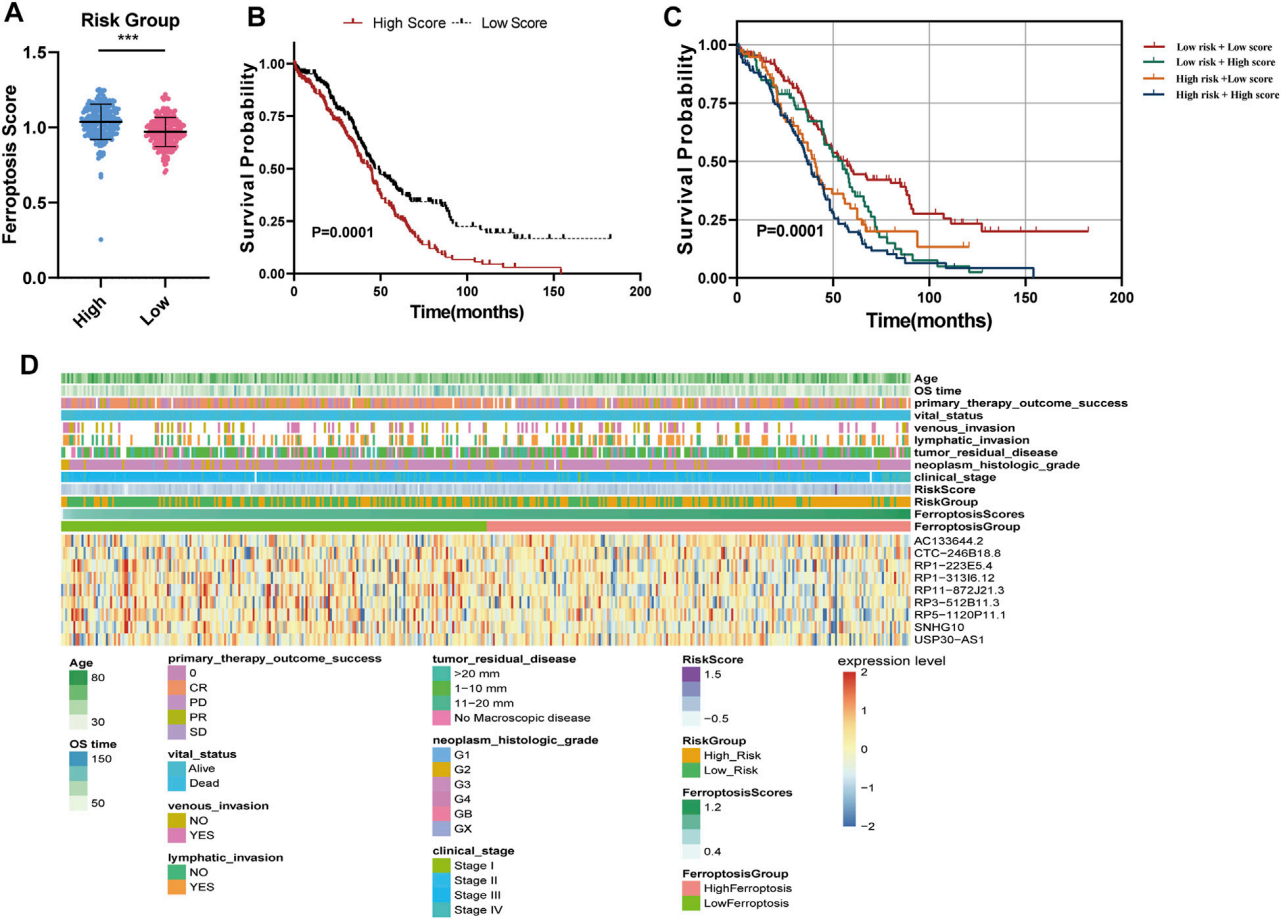
Ferroptosis score analysis **(A)** Ferroptosis scores between high and low-risk groups **(B)** The K–M survival curves of OC patients with high or low ferroptosis score **(C)** The K–M survival curves of four subgroups based on risk score and ferroptosis score **(D)** Heatmap of the associations among the expression levels of nine FRLs, risk scores, ferroptosis scores and clinical features.

We calculated the ferroptosis enrichment score of each sample. Then, we found that patients in the high-risk group had a higher ferroptosis score (Figure 5A). Patients with OC were divided into two subgroups based on the median of ferroptosis scores (FSs). The Kaplan-Meier survival curve showed that the OS of patients with the high ferroptosis score was significantly lower (Figure 5B), indicating that both high ferroptosis score and the high-risk score are risk factors (Figure 5C). Heatmap of the associations among the expression levels of nine FRLs, risk scores, ferroptosis scores and clinical features (age, stage, grade, therapy outcome, and tumor residual disease) were displayed in Figure 5D. We found that low expression of CTC-246B18.8 was associated with lower risk score, while the opposite was true for the remaining eight FRLs (Supplementary Figure S4). Low expression of CTC-246B18.8 showed lower ferroptosis score, while RP1-313I6.12, RP1-313I6.12, RP3-512B11.3, and RP5-1120P11.1 did the opposite (Supplementary Figure S5).

### Functional Pathways and Small Molecule Drugs of the Risk Groups

**FIGURE 6:**
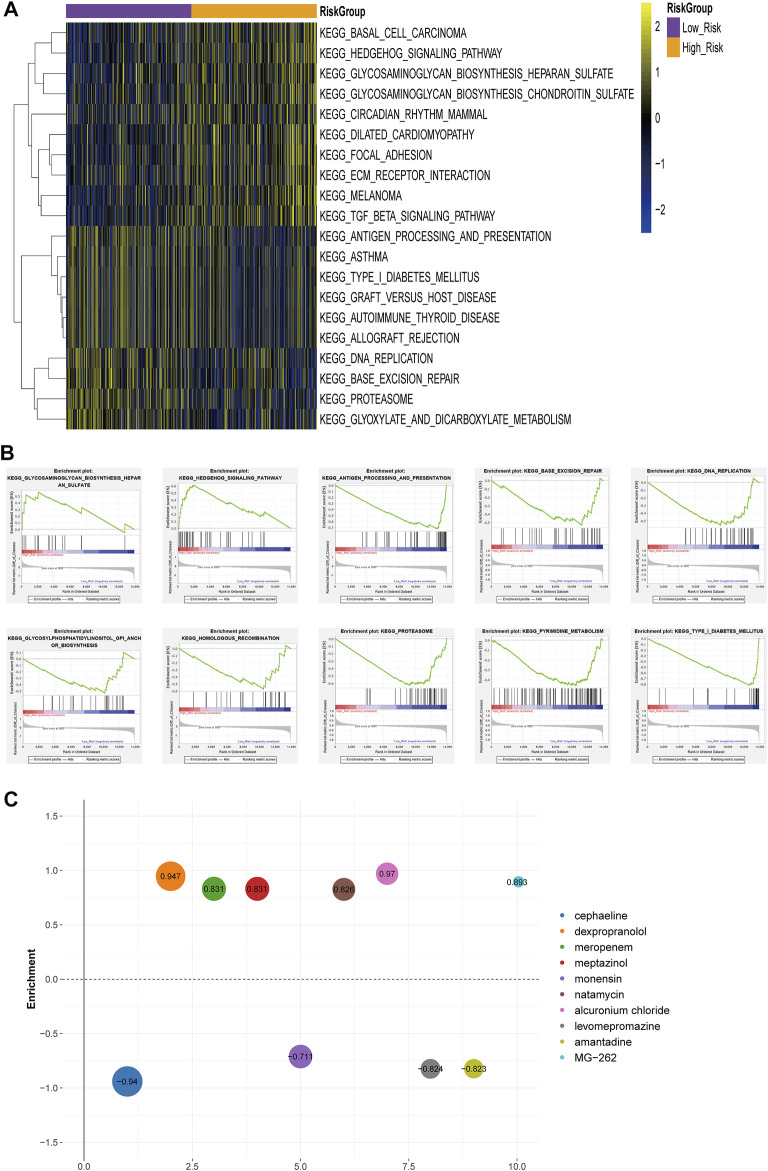
Differences in functional pathway between the risk groups **(A)** Heat map of KEGG Pathway GSVA enrichment scores **(B)** GSEA enrichment of KEGG pathway between high-risk and low-risk groups **(C)** Heatmap of small molecule drugs.

The enrichment analysis of functional pathways showed that 91 pathways showed significant differences between the two risk subgroups. The KEGG pathways were sorted according to the *p* value, and the top 10 were selected for display (Figure 6A). GSEA enrichment analysis showed that the risk groups were associated with tumor-related pathways, such as Hedgehog Signaling Pathway, Homologous Recombination, base Excision Repair, and immune-associated pathway, Antigen Processing and Presentation (Figure 6B). The differentially expressed genes (DEGs) were uploaded to CMAP database and compared with the potential drugs. The small molecule drugs were identified according to the *p* value, and the top ten were selected for display (Figure 6C).

### Immunity and m6A Regulators Analyses

**FIGURE 7:**
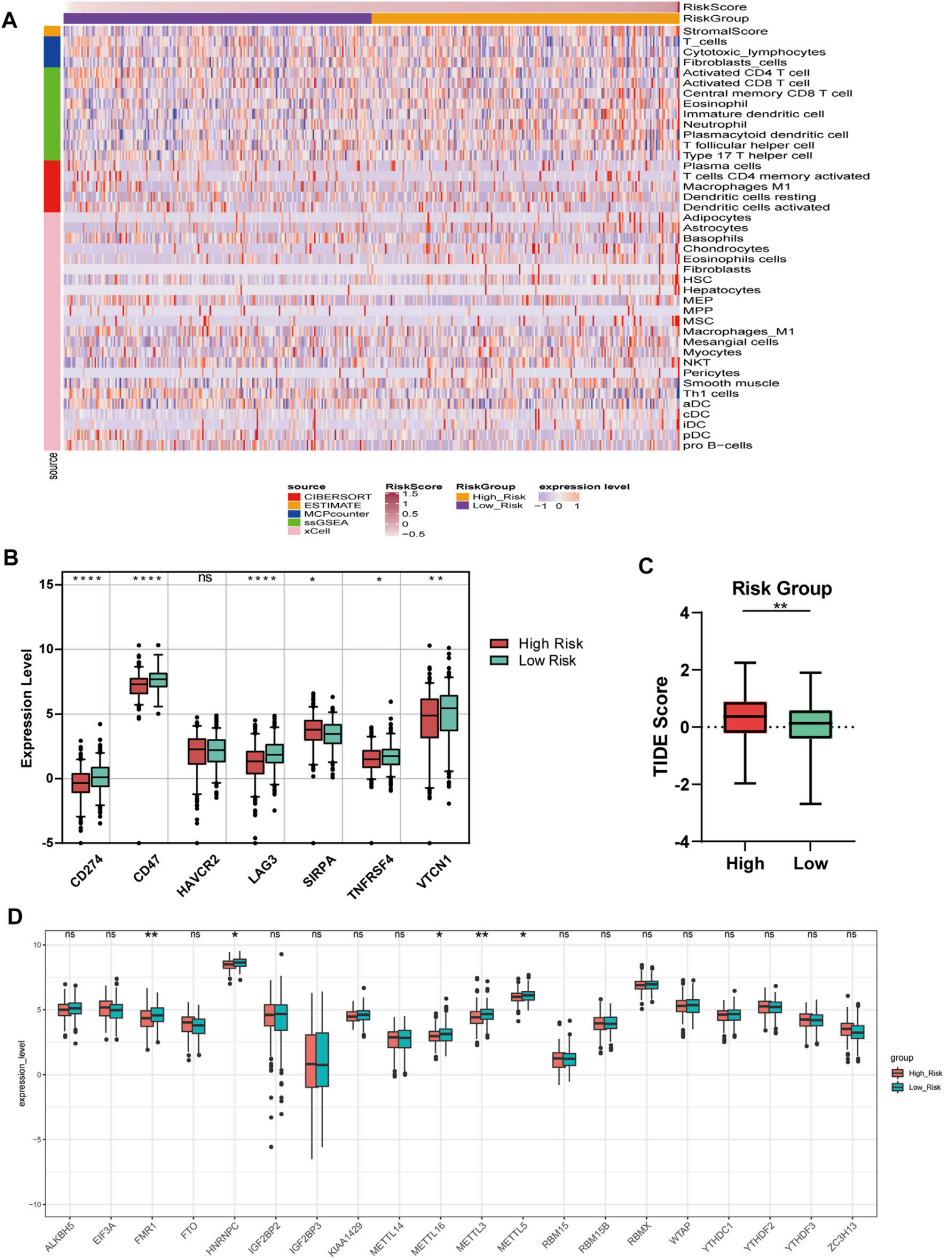
Immune and m6A analysis **(A)** Heatmap of immune microenvironment revealed that a total of 40 immune cells and stromal score had significant differences between the two risk groups **(B)** Expression of seven immune checkpoint genes between high and low-risk group. The other six genes had significant differences between the two groups (*p* < 0.05), except HAVCR2 (*p* = 0.66) **(C)** TIDE scores in the low-risk group were lower than those in the high-risk group **(D)** The expression of 20 m6A regulators between high and low-risk groups. Data are shown as means ± S.D. ns: not significant, *p < 0.05, **p < 0.01, ***p < 0.001, ****p < 0.0001.

The matrix score, immune score, estimated score, and relative infiltrate abundance of immune and stromal cells per sample were estimated based on five algorithms which were combined and shown in the heat map (Supplementary Figure S6). Wilcoxon was further used to compare the significance of each cell between the two groups. The results showed that there were statistically significant differences in interstitial fraction among 40 microenvironment cells (Figure 7A).

We compared the expression of seven checkpoint genes. The block diagram of the expression distribution of seven immune checkpoint genes (CD47, CD274, LAG3, HAVCR2, TNFRSF4, SIRPA, and VTCN1) between the two risk groups was shown in Figure 7B. The results showed that the other six genes emerged remarkable differences (*p* < 0.05), except HAVCR2 (*p* = 0.66). SIRPA had a higher expression, while the other five genes had a lower expression in the high-risk group. In Figure 7C, TIDE score of OC patients in the low-risk group was lower than that in the high-risk group, suggesting that OC patients with low RSs were more sensitive to ICB treatment.

We matched 20 m6A regulators and saw that the expression levels of FMR1, HNRNPC, METTL16, METTL3, and METL5 were significantly lower in the high-risk group. However, the differences of the remaining m6A regulators between the two groups did not reach a significant level (Figure 7D).

### Sensitivity of Chemotherapy Drugs

**FIGURE 8:**
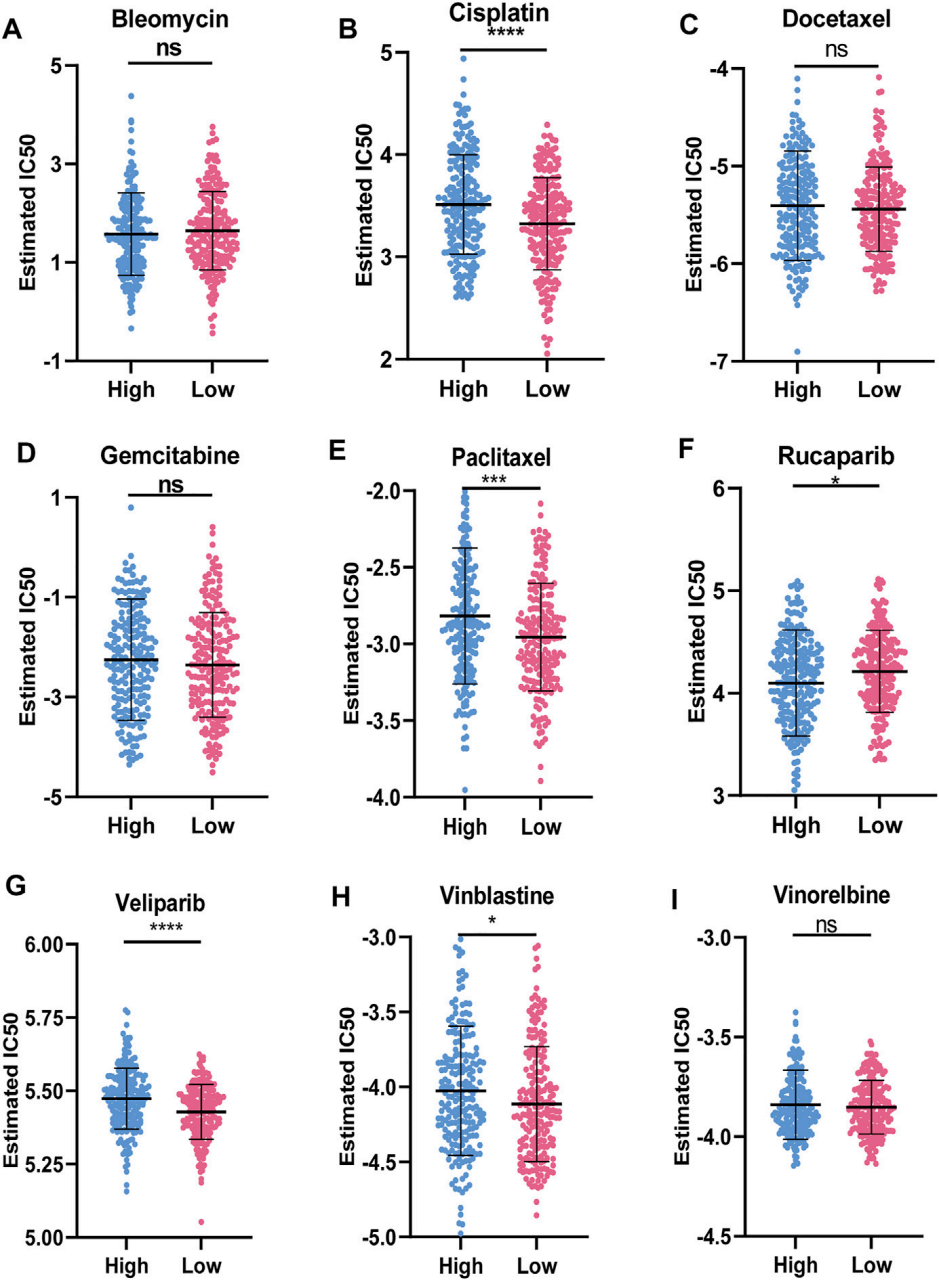
Sensitivity of chemotherapy drugs **(A-I)** Difference in the estimated IC50 levels of Bleomycin **(A)**, Cisplatin **(B)**, Docetaxel **(C)**, Gemcitabine **(D)**, Paclitaxel **(E)**, Rucaparib **(F)**, Veliparib **(G)**, Vinblastine **(H)**, and Vinorelbine **(I)**. Data are shown as means ± S.D. ns: not significant, *p < 0.05, **p < 0.01, ***p < 0.001, ****p < 0.0001.

Figure 8 showed the results of nine commonly used OC chemotherapy agents. Our data showed that the IC50 levels of Rucaparib in the low-risk group were significantly higher than that in the high-risk group. Inversely, the IC50 levels of Cisplatin, Paclitaxel, Veliparib, and Vinblastine in low-risk group were significantly lower than that in high-risk group, indicating that the OC patients in the low-risk group were more sensitive to these drugs. However, there were no significant differences in sensitivity between the two risk groups to Bleomycin, Docetaxel, Gemcitabine, and Vinorelbine.

### Network Construction

**FIGURE 9:**
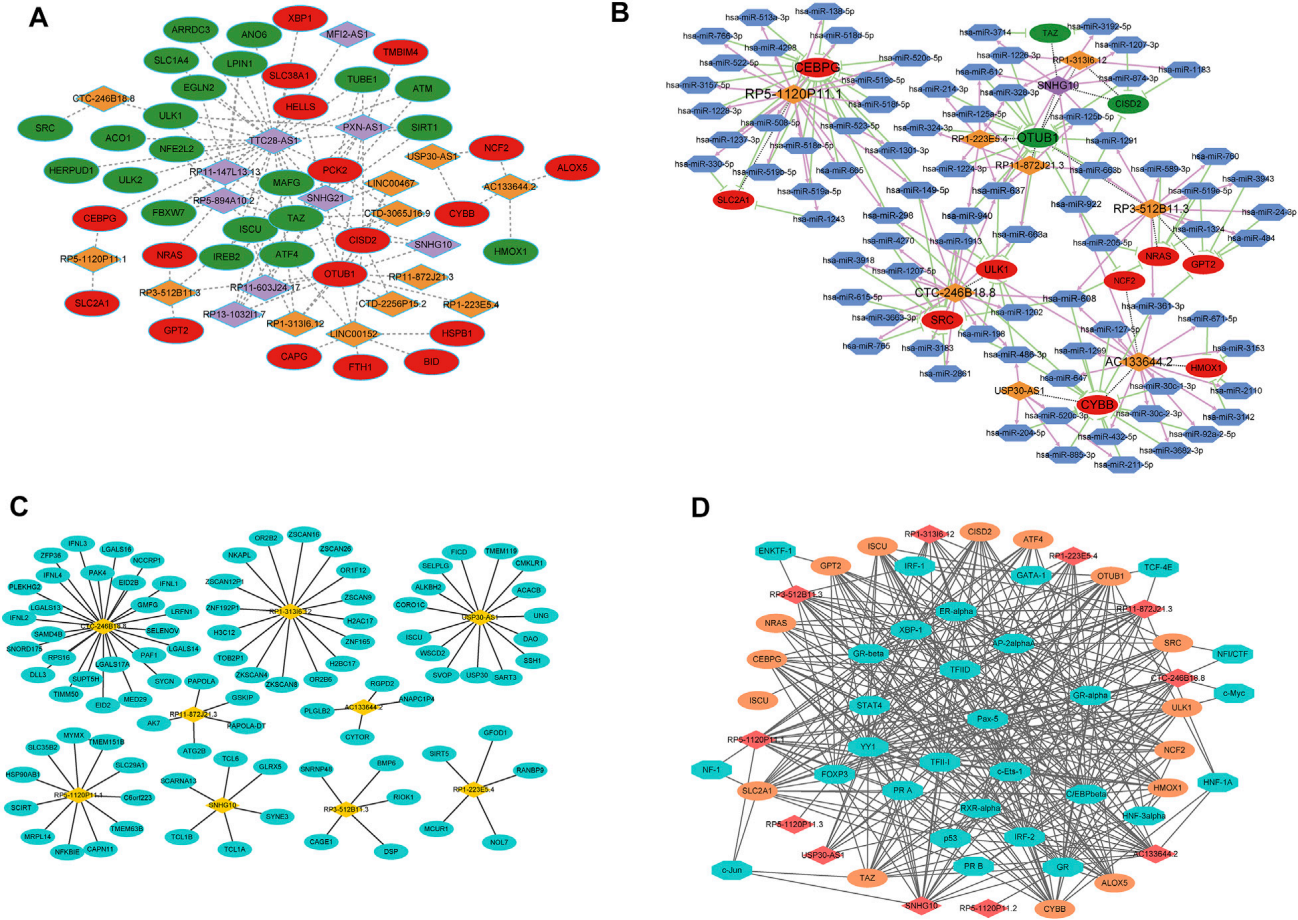
Network analyses **(A)** FRG-FRL co-expression network. Ellipses: FRGs; rhombus: FRLs **(B)** ceRNA network. Ellipses: FRGs; rhombus: FRLs; hexagon: miRNAs **(C)** lncRNA-nearby mRNA interaction networks. Rhombus: FRLs; ellipses: nearby mRNAs **(D)** Trans interaction for lncRNA-TF-mRNA relationship pairs. Rhombus: FRLs; ellipses: mRNAs; octagon: TFs.

We selected 21 FRLs with significant prognosis to fabricate the FRG-FRL co-expression network (Figure 9A). A total of 105 FRL-miRNA-FRG relationship pairs were obtained, including nine FRLs, 80 miRNAs, and 12 FRGs (Figure 9B). The 12 FRGs included: CEBPG, CISD2, CYBB, GPT2, HMOX1, NCF2, NRAS, OTUB1, SLC2A1, SRC, TAZ, and ULK1. The networks according to the cis and trans interaction were displayed in Figures 9C,D. And, we found that p53 may regulate FRGs and FRLs (CTC-246B18.8-SRC/ULK1, RP11-872J21.3-OTUB1, RP5-1120P11.1-SLC2A1/CEBPG) through trans interactions. These findings may provide a basis for understanding the regulatory mechanisms of FRLs correlated with FRGs.

## Discussion

At present, platinum and paclitaxel are the main chemotherapy drugs for OC, but the prognosis of patients with advanced OC remains poor due to drug resistance (Mikuła-Pietrasik et al., 2019). Ferroptosis, as a newly discovered non-programmed cell death mode, is closely related to a variety of physiological and pathological processes (Li et al., 2020a). The iron dependence of tumor tissue is a potential therapeutic target for tumors (Shen et al., 2018; Liang et al., 2019). The use of iron chelating agents and the action of cytotoxic drugs such as TFR can inhibit the growth of cancer cells to a certain extent and improve their sensitivity to chemotherapy drugs by depriving iron or changing the metabolic ways of iron uptake and transfer in cancer cells (Moussa et al., 2019). Hence, the further studies on the signaling pathway and induction mechanism of ferroptosis are worth doing in clinical, and metal antitumor drugs may replace platinum chemotherapy drugs as a new generation of anticancer drugs to improve the prognosis.

Recently, continuous studies confirm that lncRNA is involved in the regulation of ferroptosis in tumor cells. It is unveiled that LINC00336 accelerated tumor formation and inhibited ferroptosis of lung cancer through the LSH/ELAVL1/LINC00336 axis (Wang et al., 2019b; Wang et al., 2020b). In hepatocellular carcinoma, the chemical stimulation produced by ferroptosis inducer Erastin can up-regulate the expression of lncRNA GABPB1-AS1 and prolong its half-life (Qi et al., 2019). In lung cancer, lncRNA NEAT1 was proved to be a regulator of ferroptosis sensitivity (Wu and Liu, 2021), and lncRNA MT1DP modulated erastin-induced ferroptosis via miR-365a-3p/NRF2 axis (Gai et al., 2020). LINC00618 was confirmed as accelerant of ferroptosis via reducing the expression of SLC7A11 in leukemia (Wang et al., 2021). Regrettably, researches of FRLs are still scarce.

As for OC, lncRNA-based prognostic signatures have been reported in previous investigations (Li et al., 2021; Pan and Bi, 2021). There are also lncRNA-based prognostic signatures for other cancers, such as breast cancer (Li et al., 2020b; Ma et al., 2020), glioma (Lin et al., 2020), bladder urothelial carcinoma (Sun et al., 2020), colorectal cancer (Sun et al., 2020; Zhang et al., 2021), esophageal cancer (Liu et al., 2021). To our knowledge, our study is the initial to identify and comprehensively analyze the ferroptosis-related lncRNAs signature for OC, providing a promising strategy for guiding individual therapy and improving prognosis prediction, and has important clinical implication. We also provide an effective quantitative method of FRL-related nomogram for predicting the OS in OC patients in combination with clinical factor. In addition, all the prognostic models reported are different, and no unified prognostic model has been applied in clinical practice. In this study, a new prognostic risk model was proposed, and its clinical applicability deserves further study.

Additionally, a total of 40 microenvironment cells, six immune checkpoint genes, and five m6A regulators (FMR1, HNRNPC, METTL16, METTL3, and METTL5) showed different levels between the two groups. Research reported that activated CD8^+^ T cells during immunotherapy could heighten the ferroptosis specific lipid peroxidation in tumor cells and, conversely, activation of ferroptosis are conducive the antitumor effect of immunotherapy (Wang et al., 2019c). It has also been found that immunotherapy could sensitize tumor cells to treatment by regulating ferroptosis of tumor cells (Lang et al., 2019). In addition, PD-1 antibodies could produce an immunogenic microenvironment and induce ferroptosis of tumor cells, and tumor antigens released after cell death in turn promote the immunogenicity of the microenvironment, suggesting that the cyclical synergistic effect of ferroptosis and immune regulation achieves effective antitumor activity *in vivo* (Zhang et al., 2019). A for the relationship between ferroptosis and m6A modification, the m6A reader YTHDC2 serving as ferroptosis inducer was proved to regulate SLC3A2 in lung adenocarcinoma (Ma et al., 2021). It was shown that miR-4443 inhibited FSP1-mediated ferroptosis via m6A writer METLL3 (Song et al., 2021). All these provide a theoretical basis for us to study the specific mechanism of ferroptosis in tumors. The occurrence and development mechanism of malignant tumors is very complicated. We hope to explore the molecular mechanism of iron disease (through m6A modification or immunity) through further research, so as to improve the efficacy of immunotherapy for malignant tumors.

The signature we identified and validated containing nine FRLs with prognostic value, which have never been reported in OC. Among the nine FRLs, USP30-AS1 was identified as autophagy-related lncRNA in bladder urothelial carcinoma (Sun et al., 2020), OC (Meng et al., 2020), and bladder cancer (Wan et al., 2021), immune-related lncRNA in cervical cancer (Chen et al., 2020), and epithelial–mesenchymal transition-related lncRNA in bladder cancer via bioinformatic analysis. In the literature mentioned, USP30-AS1 has been identified as a protective factor in cancers which is consistent with our study. SNHG10 has been confirmed as a malignant gene in hepatocellular carcinoma (Lan et al., 2019; Zhu et al., 2019), gastric cancer (Zhang Y et al., 2021; Yuan et al., 2021), glioma (Jin et al., 2020), osteosarcoma (He et al., 2020; Zhu et al., 2020), and acute myeloid leukemia (Xiao et al., 2021), while it was certified as a suppressor in non-small cell lung cancer (Zhang et al., 2020b) and our study. Hence, SNHG10 may play a dual role in malignancy.

We further explored the differences in respond to ICB therapy and chemotherapy drug between risk groups. Our result revealed that the patients of OC with lower RSs were more sensitive to ICB therapy, Cisplatin, Paclitaxel, Veliparib, and Vinblastine, while the patients were more sensitive to Rucaparib in the high-risk group. Findings of our study uncovered FRLs-based risk models identified potential biomarkers and therapeutic targets.

There are still some limitations. Firstly, there are only 30 OC patients in our cohort did not have OS, hence future studies need more time and samples for follow-up. Secondly, the number of OC samples in TCGA is very limited, so more independent data sets are needed to verify the risk model we identified. However, the tumor risk prediction model plays an important role in the treatment of tumor, and its value is considerable. Clinicians can carry out more accurate treatment according to the model, and also estimate the prognosis of patients according to the risk model, and develop more personalized treatment plans.

## Conclusion

In conclusion, the risk model identified and validated based on nine ferroptosis-related lncRNAs is an independent prognostic factor. By comprehensive analysis, finding of our study revealed potential biomarkers and therapeutic targets for FRL-based risk models.

## Data Availability

The datasets presented in this study can be found in online repositories. The names of the repository/repositories and accession number(s) can be found in the article/Supplementary Material.

## Abbreviations

FRG, Ferroptosis-related gene; FRL, Ferroptosis-related lncRNA; FS, Ferroptosis score; GO, Gene ontology; GRCh38, Genome Reference Consortium Human Build 38; GSEA, Gene Set Enrichment Analysis; HR, Hazard ratio; K-M, Kaplan–Meier; LASSO, Least absolute shrinkage and selection operator; m6A, N6-methyladenosine; OC, Ovarian cancer; OS, Overall survival; PCC, Pearson correlation coefficient; RS, Risk score; TCGA, The Cancer Genome Atlas.
