# Supplementary figures and images for: Bioinformatic Analyses of the Ferroptosis-Related lncRNAs Signature for Ovarian Cancer
Source: Front Mol Biosci. 2022 Jan 18;8:735871. doi: 10.3389/fmolb.2021.735871 (PMC8807408; doi:10.3389/fmolb.2021.735871)

**A AC133644.2**

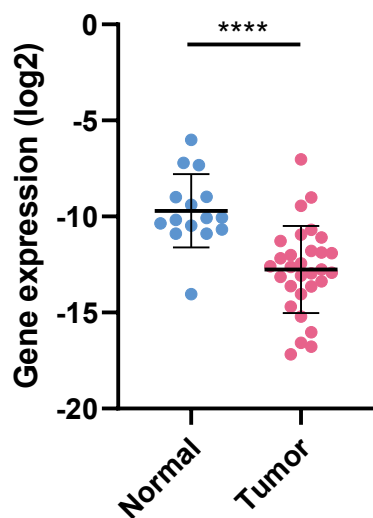

**B CTC-246B18.8**

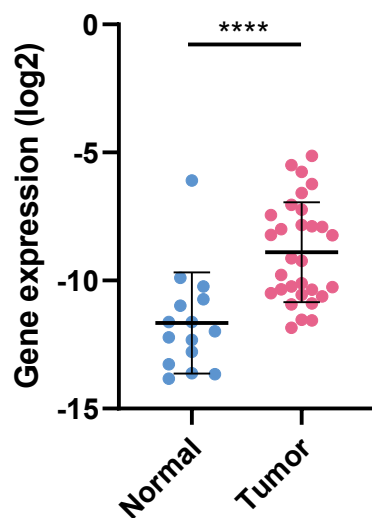

**C RP1-223E5.4**

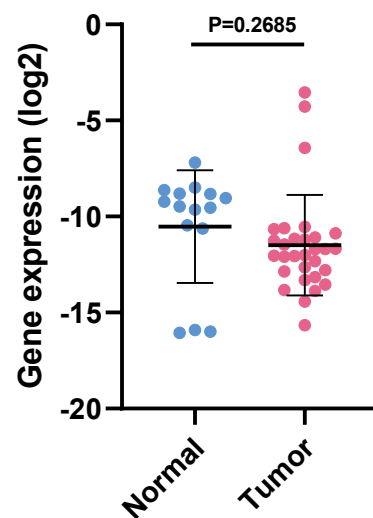

**D RP1-313I6.12**

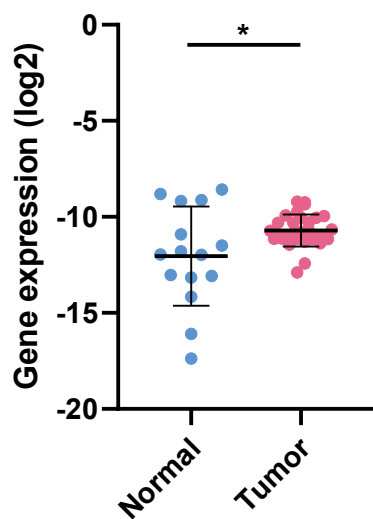

**E RP11-872J21.3**

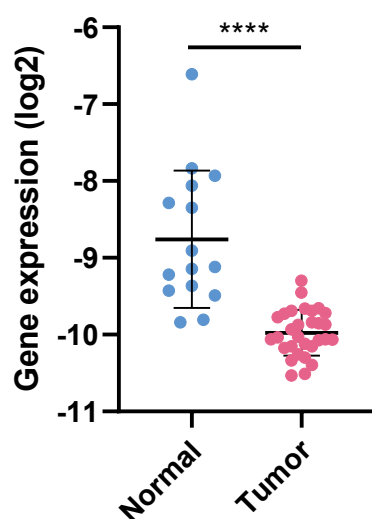

**F RP3-512B11.3**

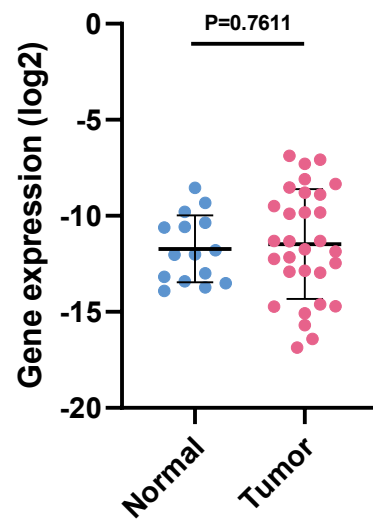

**G RP5-1120P11.1**

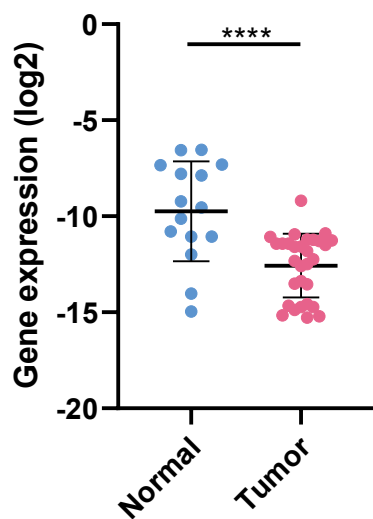

**H SNHG10**

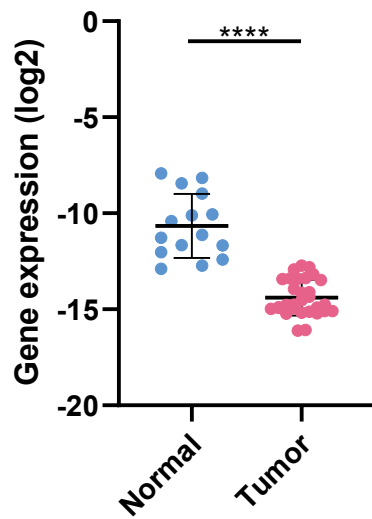

**I USP30-AS1**

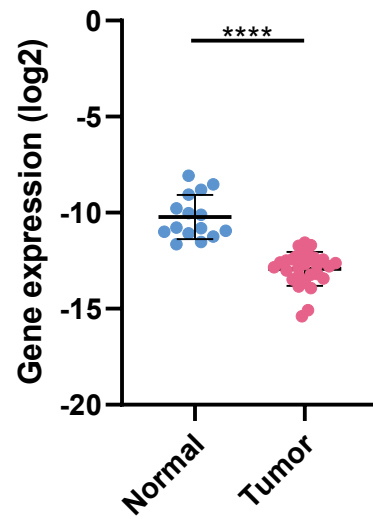

Supplement: Supplementary file 1 [file DataSheet2.PDF]

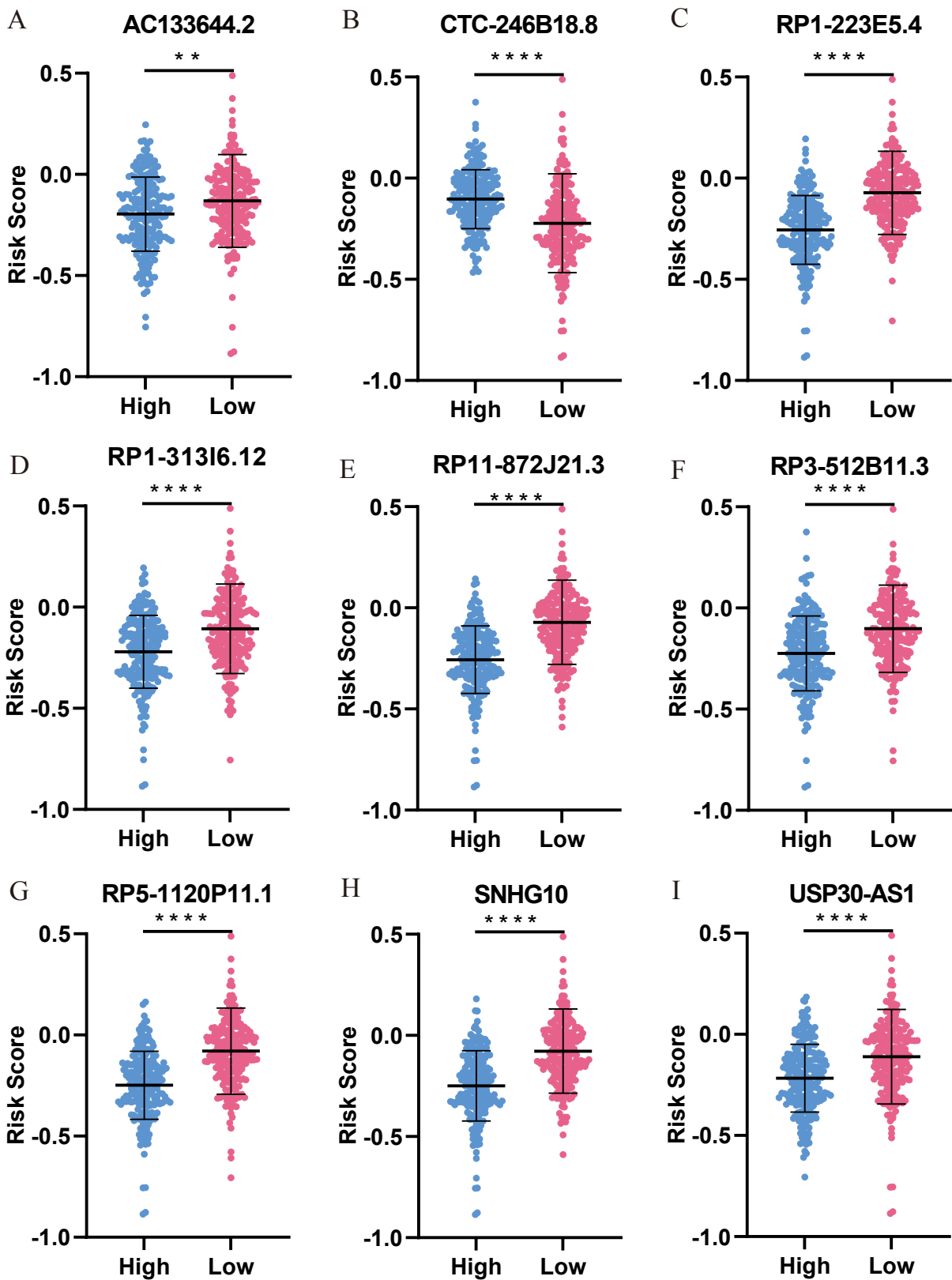

Supplement: Supplementary file 2 [file DataSheet4.PDF]

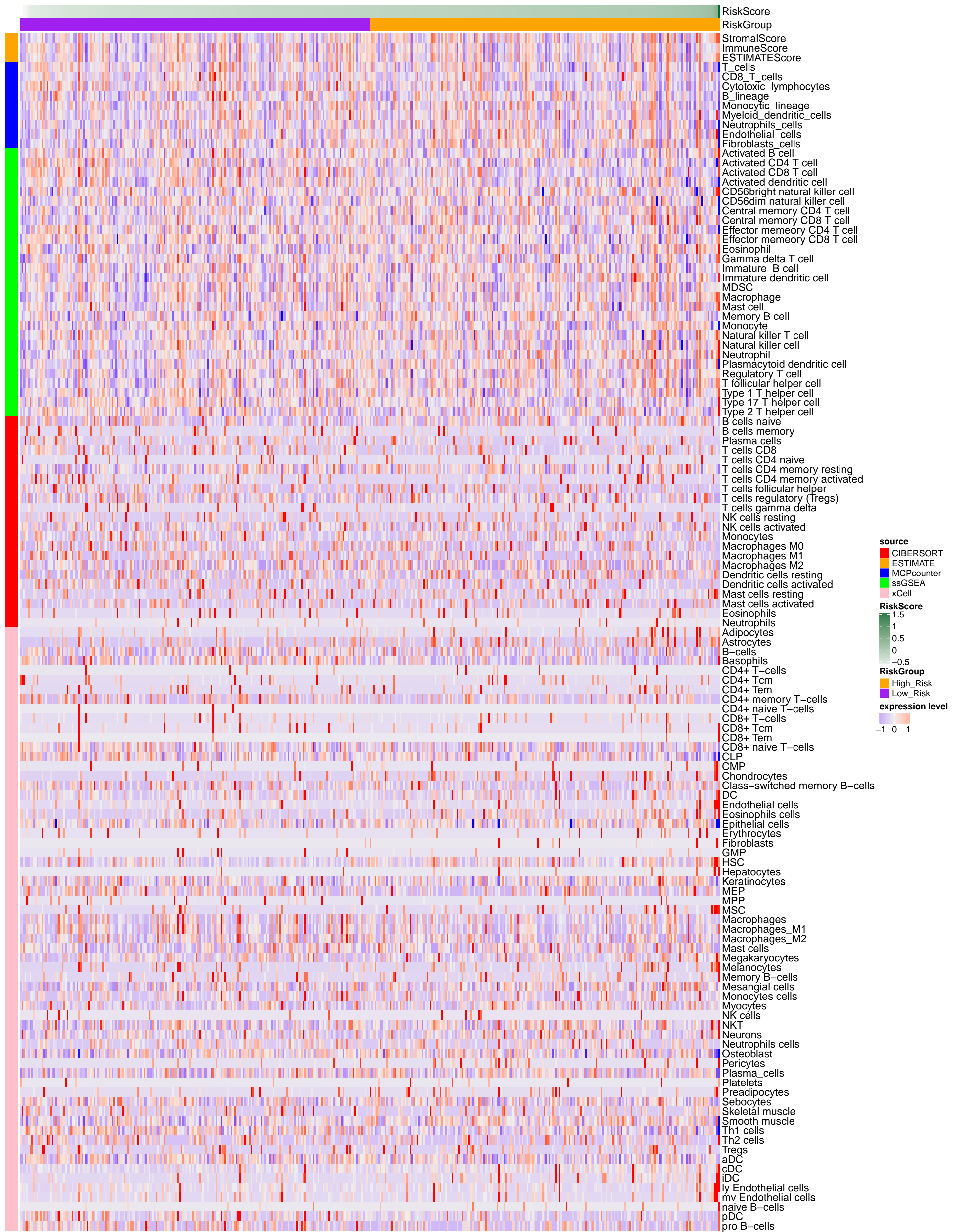

source

Supplement: Supplementary file 3 [file DataSheet6.PDF]

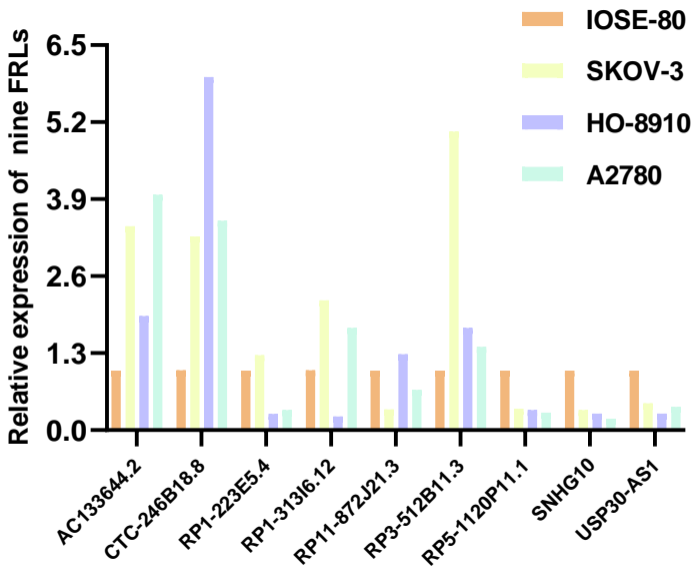

Supplement: Supplementary file 4 [file DataSheet3.PDF]

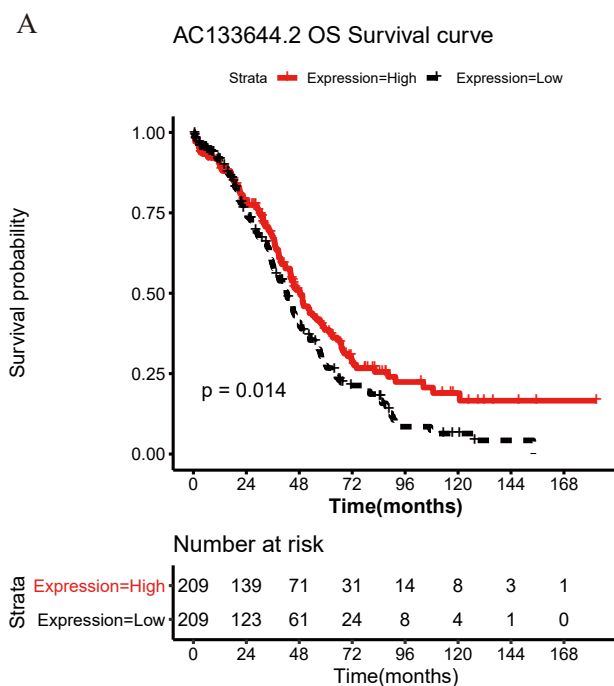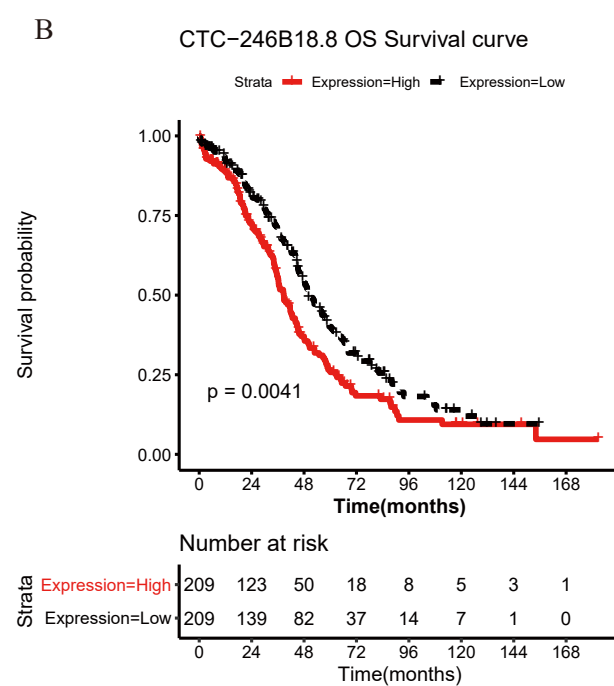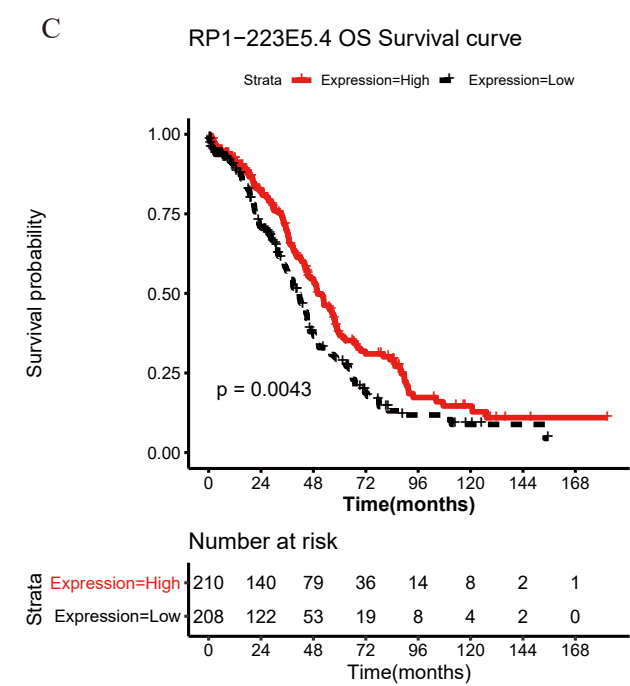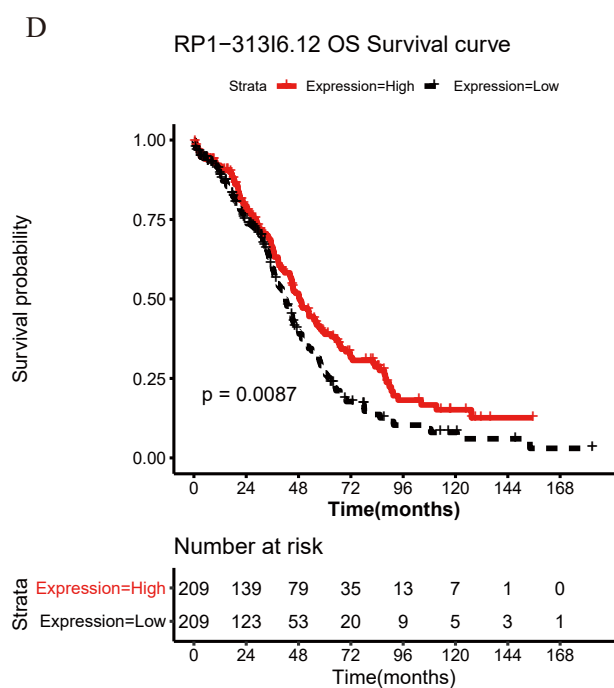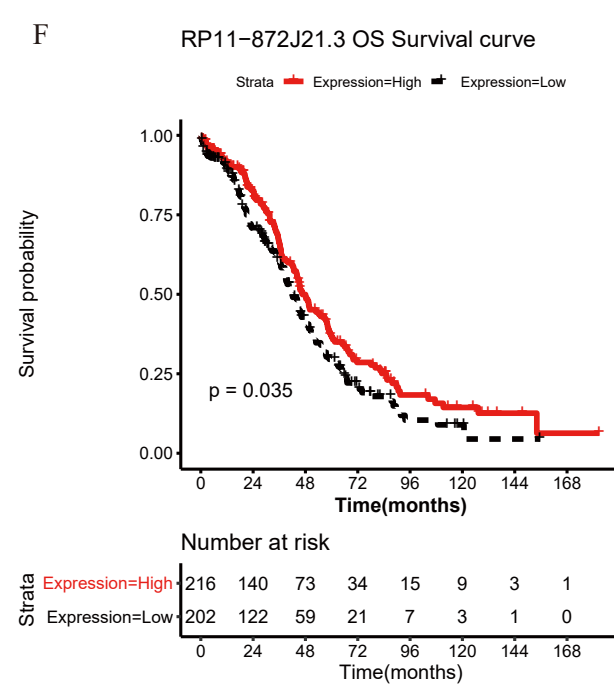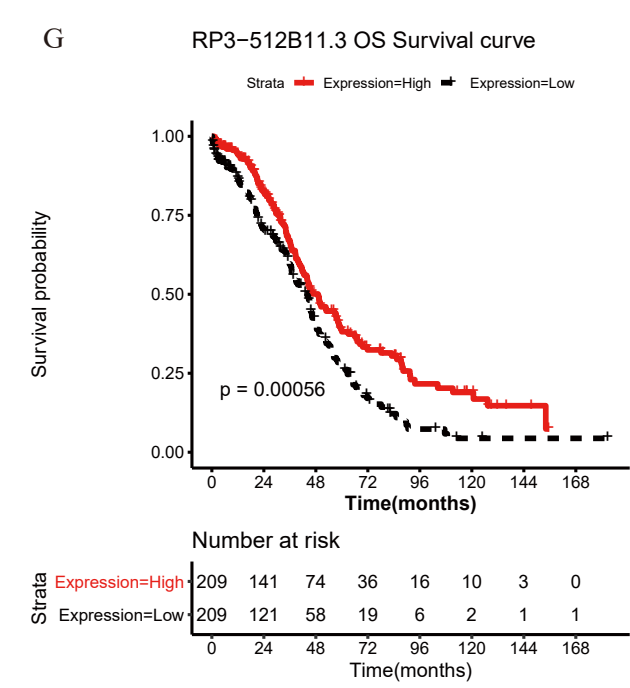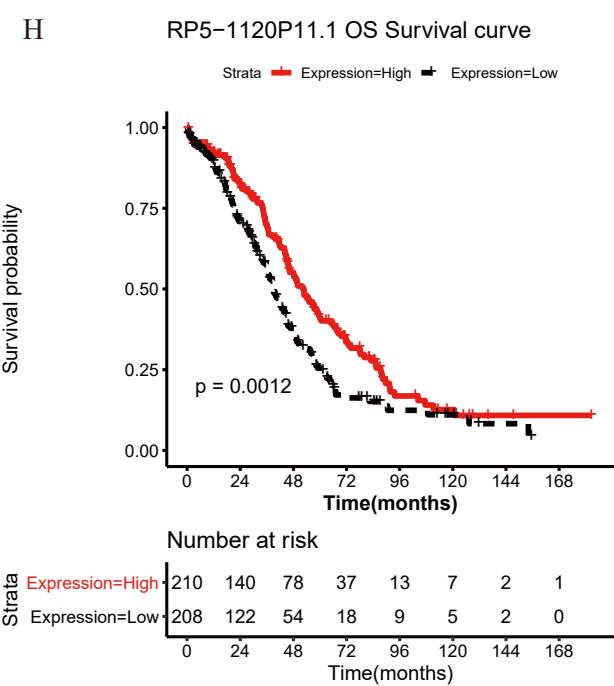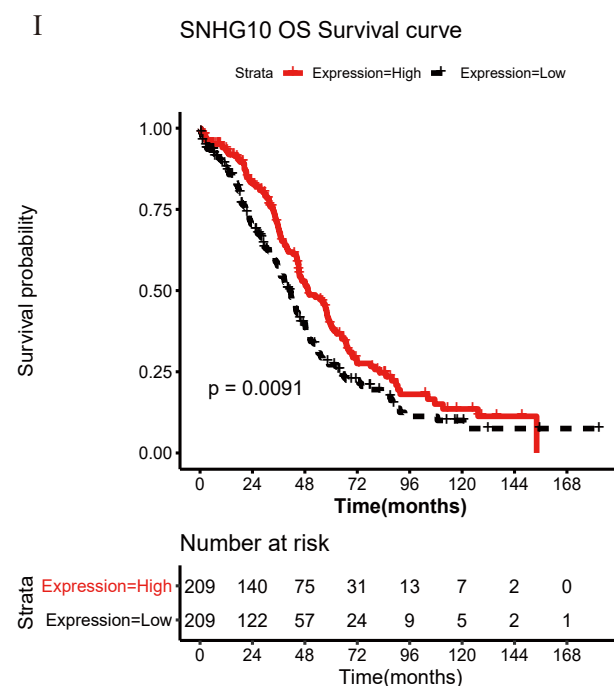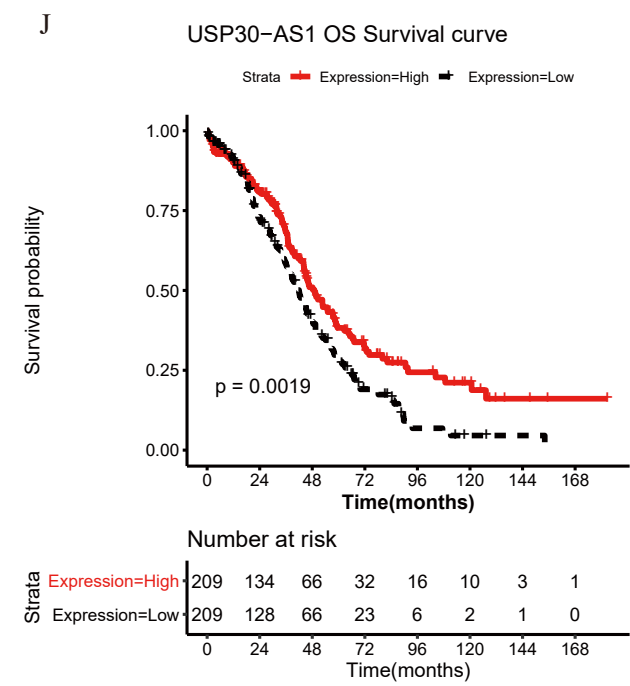

Supplement: Supplementary file 5 [file DataSheet1.PDF]

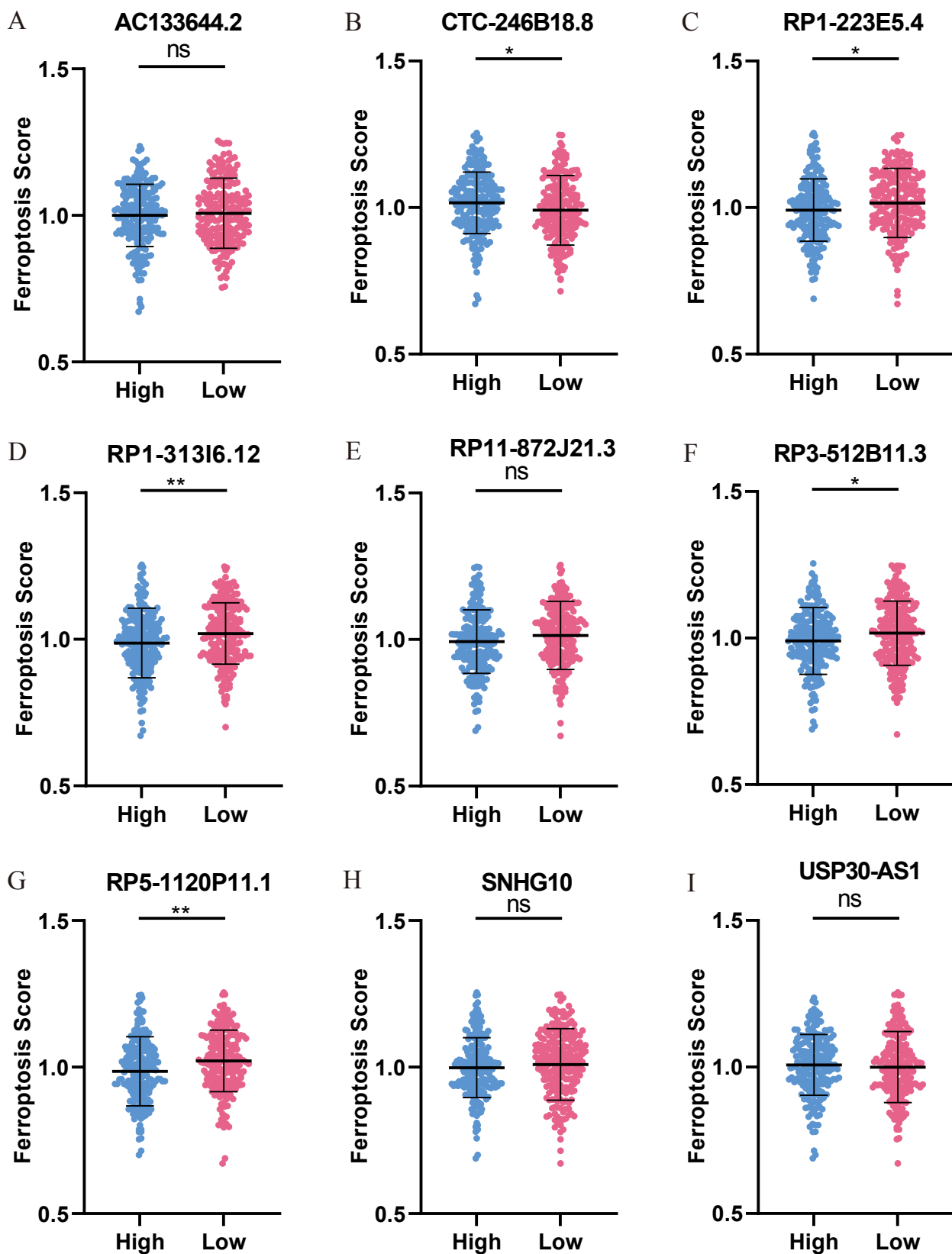

Supplement: Supplementary file 6 [file DataSheet5.PDF]
